# The Mesoscopic Electrochemistry of Molecular Junctions

**DOI:** 10.1038/srep18400

**Published:** 2016-01-13

**Authors:** Paulo R. Bueno, Tiago A. Benites, Jason J. Davis

**Affiliations:** 1 Institute of Chemistry, Physical Chemistry Department, Univ. Estadual Paulista (São Paulo State University, UNESP), Nanobionics group, CP 355, 14800-900, Araraquara, São Paulo, Brazil; 2 Department of Chemistry, University of Oxford, South Parks Road, Oxford OX1 3QZ, UK

## Abstract

Within the context of an electron dynamic (time-dependent) perspective and a voltage driving force acting to redistribute electrons between metallic and addressable molecular states, we define here the associated electron admittance and conductance. We specifically present a mesoscopic approach to resolving the electron transfer rate associated with the electrochemistry of a redox active film tethered to metallic leads and immersed in electrolyte. The methodology is centred on aligning the lifetime of the process of electron exchange with associated resistance and capacitance quantities. Notably, however, these are no longer those empirically known as charge transfer resistance and pseudo-capacitance, but are those derived instead from a consideration of the quantum states contained in molecular films and their accessibility through a scattering region existing between them and the metallic probe. The averaged lifetime (*τ*_*r*_) associated with the redox site occupancy is specifically dependent on scattering associated with the quantum channels linking them to the underlying metallic continuum and associated with both a quantum resistance (*R*_*q*_) and an electrochemical (redox) capacitance (*C*_*r*_). These are related to electron transfer rate through *k* = 1/*τ*_*r*_ = (*R*_*q*_*C*_*r*_)^−1^. The proposed mesoscopic approach is consistent with Marcus’s (electron transfer rate) theory and experimental measurements obtained by capacitance spectroscopy.

Mesoscopic physics (coined^1^ as the study of physical/chemical systems in the intermediate size range sitting between nanometric and macroscopic) is a relatively young branch of physical science and is largely concerned with questioning the operation of quantum mechanical rules as one accesses the classical macroscopic regime^2^. Mesoscopic analyses thus seek to bridge between the molecular and man-made structures and are highly relevant to the interface between these across physics, chemistry, electronics, optics and technologies generally. The analysis of molecular capacitance and conductance is a mesoscopic problem sitting exactly within this regime^2^,^3^.

A theoretical consideration of electron transfer and electron transport, in its multitude of guises, underpins our understanding of some of the most fundamental biological and technological processes, and has been an intensely rich and active field for more than five decades^4^,^5^,^6^,^7^,^8^,^9^. In the field of molecular electronics, we consider charge movement between electrode (generally metallic) states through an intervening molecular pathway (see Fig. 1a). In this configuration conductance should be related to the ease with which electrons can traverse the molecular pathway as considered in terms of a transmission probability. In terms of the molecular electronic structure influence on transmission probability, electron transport is now readily accessible to computational treatment within a non-equilibrium Green’s function (NEGF) approach^10^ in combination with a density functional theory (DFT)^11^ based description of the junction (i.e. the understanding of electron dynamics has advanced quickly thanks to the incorporation of many-body physics^11^), namely its leads and scattering regions (see Fig. 1). Experimental comparisons are, of course, possible and can be strikingly good^12^. An improved alignment with experiment remains, though, a formidable challenge for DFT, albeit one that enables an atomistic interpretation of experiments such as those within the confines of an electrochemical scanning tunnelling microscopy (STM)^13^. Fundamental to DFT is a consideration of molecules as specific *N*-electron systems^14^.

In the field of molecular electrochemistry, on the other hand, the analyses of electron transfer through a junction formed between a (commonly) metallic probe and a molecule (Fig. 1b) containing electronically addressable quantum states, be the latter surface attached or solution phase, is made by embedding this junction in an electrolytic medium. The process of electron exchange between metallic states and those quantum states in the molecule is itself that through the intervening molecular dielectric^12^. Observations are at least qualitatively understood when viewed through classic macroscopic treatments^4^ and our understanding of electron transfer/transport continues to support research activities and developments across a vast range of fields.

Charge transfer kinetics/electron transfer rates in electrochemistry are well-known to obey, at least approximately, an exponential activation law (known as Tafel’s equation^4^) for the relationship between faradaic current (the current exclusively associated with redox change) and the electrode potential^4^,^15^, *V* = (*V*_*a*_ − *V*_*eq*_), where *V*_*a*_ is the electrode potential (bias) applied with respect to *V*_*eq*_, i.e. the potential where redox reactions are in thermodynamic equilibrium (the so called half-wave potential for reversible electrochemical systems). This activation law can thus be expressed as


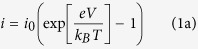


or either by its electrochemistry^4^ equivalent as


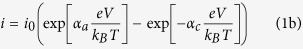


where *e* is the elementary positive charge, *k*_*B*_ is the Boltzmann constant, *T* is the absolute temperature, *α*_*a*_ and *α*_*c*_ are the so-called (dimensionless) anodic and cathodic electron transfer coefficients^4^, respectively. *i*_0_ is the net current occurring or measured at the interface. Note in Eqn. (1*a*) that the current increases at negative bias and the term (–1) ensures that the net current is zero/null in the absence of an applied electrochemical bias, *eV*, that is modulated by 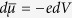 (where 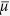 is the electron chemical potential in the electrode), as exemplified in Fig. 1b. Note also that Eqn. (1) represents the current *versus* potential phenomenology occurring in metal-semiconductor junctions, which, depending on the differences between the work functions of the metal and that of semiconductor, is representative of a Schottky diode^16^. Eqn. (1*b*) is the so-called Butler-Volmer equation which is extensively used to study electron transfer kinetics in single probe (see Fig. 1) molecular junction configurations. At electrochemical equilibrium (*V*_*a*_ ~ *V*_*eq*_), the forward (*i*_+_) and backward (*i*_−_) direction currents are equivalent and net current *i*_0_ thus vanishes^4^. It is important to note that in electron transfer kinetics the anodic and cathodic coefficients in Eqn. (1*b*) are related *α*_*a*_ = 1 − *α*_*c*_ such that now both Eqn. (1*a*) and (1*b*) lead to the same approximations at electrochemical equilibrium (small over-potential), i.e. *i* = *i*_0_*eV*/*k*_*B*_*T*. This small over-potential region is termed the *Ohmic region* and is a region of potential where the associated resistance/conductance (see below) will be a constant value characteristic of the electrochemical junction through *i*_0_.

The measurement of *i*_0_ is possible within a time/frequency dependent experimental setup offered by Electrochemical Impedance Spectroscopy^4^. Within this, the well-known charge transfer resistance, *R*_*ct*_, defined as the reciprocal of the infinitesimal variation of the current with respect to the potential, is a key parameter. Following Ohm’s law, from Eqn. (1) we obtain *R*_*ct*_ = (*di*/*dV*)^−1^ = *k*_*B*_*T*/*i*_0_*e* (at the *ohmic region*) as the resistance to transfer one single electron from metallic to molecular states at the junction Fermi level (the reciprocal of this quantity is *G*_*ct*_ = *i*_0_*e*/*k*_*B*_*T*, the conductance associated with the electrochemical charge transfer process). *R*_*ct*_ is then predicted to be null for a zero temperature limit equivalent to the macroscopic resistance as defined by the electron gas model^3^. Quantized (or mesoscopic) characteristics are, thus, not expected to be resolved in Eqn. (1) or *R*_*ct*_ (as they represent classical macroscopic quantities).

Regardless of whether the experimental operation is molecular electronic (Fig. 1a) or molecular electrochemical (Fig. 1b) in configuration, the atomic/molecular scale (quantum mechanical effects) are, of course, central. Experimental analyses of electron transport across molecular structures (bridges between lead contacts as exemplified in Fig. 1a) are essentially mesoscopic (meaning phenomena occurring at a sufficiently small scale for quantum mechanical characteristics to have a detectable influence over measured physical properties), for example. It is well-known that conductance can be formulated in terms of a quantum of conductance and that there is an associated molecular scale resistance. At low temperatures the transport regime associated with Fig. 1a is phase coherent or primarily ballistic at very short distances (meaning elastic scattering prevails); thermal broadening does not occur and electronic states remain spectrally discrete. The application of scattering theory to *direct current* (DC) electrical conduction has been successfully verified multiple times now^14^,^17^,^18^,^19^,^20^,^21^,^22^ within two probe (lead) configurations and ultimately relates the transmission probabilities of the occupied one-dimensional sub-bands or quantum wells (see SI. document section 2.1 for more details) to the DC conductance through *G* = (2*e*^2^/*h*)*T*_*r*_(*t*^†^*t*) (Landauer formula for multi-channel conductance)^10^,^23^,^24^, where *e* is the elementary charge, *h* is the Planck constant and *t* is the transmission matrix and *T*_*r*_ is the trace matrix operator. *t* predicts a quantised conductance (*G*_0_ = 2*e*^2^/*h*, about 7.75 × 10^−5^ Ω^−1^), i.e. the quantum of conductance for a quasi-one dimensional constriction when the magnitude of the transmission coefficient for each channel is unity (ballistic motion). More generally 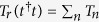, where *T*_*n*_ is the transmission coefficient of transmission channel *n*. The channels herein represent the quantum mechanical probabilities of the electron exchanging energy states communicating. The electron transmittance obviously depends on the physical characteristics of the scattering region (Fig. 1a).

Molecular electrochemical experiments, in contrast, are performed within a single-probe configuration (Fig. 1b), where DC conductance/resistance is absent (experimentally represented by the inset of Fig. 2a), but *alternate current*, AC, or time-dependent conduction/transport analysis is measureable and has an associated capacitance, *C*_*r*_ (proportional to the electrochemical density of states of the molecular monolayer junction^14^,^22^,^25^,^26^,^27^ – see SI. section 1.1 and 1.2. for more information on the experimental methodology by which this is measured/obtained). It is important to note that the electrochemical density of states of a molecular/metallic junction (as obtained through electrochemical capacitance, *C*_*r*_) represents the amount of redox states available to be oxidized or reduced through electron exchange with the underlying metallic probe. These states are maximized at the electrochemical equilibrium where the electrochemical potential of the electrons in the metal probe, 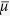, equates to those of molecular electroactive layer, *E*_*r*_. This redox DOS is different to the Marcus-Gerisher^4^ density of reduced and oxidized states as applied to electroactive molecular species in solution phase; in this case the density of states is nuclear (vibronic DOS). The electronic redox density of states we are refer to herein is apparent only on confining quantised and accessible electronic states to a metallic probe and not for states free in the bulk solution^14^. In such molecular junctions, the redox charging that accompanies electrochemistry specifically has a magnitude quantified by its associated redox capacitance.

This capacitive element depends, in a significant way, on the properties of the mesoscopic conductor and its nearby environment/gates as we have demonstrated previously^22^,^25^. In these room temperature experiments, thermal agitation blurs the resolved electronic states into a continuous density of state (a Gaussian-shape with the centre located at energies where 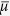 equates to *E*_*r*_)^25^,^27^,^28^ that constitutes the accessible redox density of states (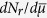) of the molecular film^14^. These molecular layers contain, of course, quantum states, connected to an electron reservoir (electrode) and exchanging electrons in a process energetically driven by 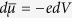 and capacitively coupled (through *C*_*r*_). Within the nomenclature utilised herein one may note that 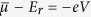. On taking a derivative of 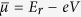 with respect to the potential (*V*) and considering *E*_*r*_ (the Fermi level or the electrochemical potential of the electrons in the molecular layer) as constant, we arrive at 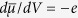 or 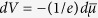.

In a “conventional” electronic conductor (sandwiched by two metallic probes) the potential difference (*dV*) between the two metal probes is given by the electric field divided by the probe separation length. On the other hand, in an electroactive molecular layer junction (assuming that the probed electroactive molecular layer interface passes electrons but not ions), the same measurement determines the gradient of the electron free energy per unit charge, which has units of electric field only. Therefore, the potential difference between metallic probe and redox centers in a molecular layer, *dV*, is simply the difference in electron free energy per unit of charge between those two spatial points (approximately the molecular length or the distance between the redox and metallic centers) such that 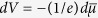 is directly proportional to the Gibbs free energy, 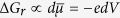, of the electrochemical process. It can also be shown thus that the electron current density is thus proportional to the gradient of the local cell potential, *dV*, and/or Δ*G*_*r*_.

Herein we are interested in the current driven through this molecular structure in response to a voltage oscillation in the electrode, from where the time-dependent (dynamic) aspects of electron transfer/conduction can be analysed. The phase or time lag between perturbation (voltage) and response (current) is specifically related to the charge storage (electrochemical capacitance) concepts introduced by Büttiker^29^. As previously mentioned, in terms of a molecular electronics approach, this is not resolved in (classical) time-independent (or DC) analyses such as those typified in Fig. 1a because the electron flux (current) between the metallic probes is null (see Fig. 1d and related legend) leading to a zeroed transmittance probability (in the Landauer formulation) and a reflectance as unity at larger times (DC conductance). In other words, in molecular electronics, a voltage (energetic) difference between the metallic probes (as indicated in Fig. 1c) is a requirement in seeking information from the scattering region and under conditions of an absence of net electron flux no information can be obtained in a DC approach. On the other hand, *alternate current* (AC) and *perturbing methods*, also known as time-dependent analyses, facilitate a control and resolution of the time domain associated with any contributing electronic mobility (even in the a situation like that in Fig. 1b where DC conductance is not possible) and specifically provides information on scattering from molecular components bridging between redox states and a single metallic continuum. Electroactive self-assembled molecular layers/junctions thus constitute a single probe means of analysing molecular conductance and/or electron transfer within an energetic coupling between metallic states and those of the molecular layer at the resonance frequency, i.e., when the perturbing frequency is equivalent to the reciprocal of the time scale of the associated electrochemical rate constant. The electrochemical processes under analysis here concern the redox pair given by [AuS-(CH_2_)_11_-Fc]/[AuS-(CH_2_)_11_-Fc^+^] where only the ferrocene component displays accessible redox chemistry (Fc/Fc^+^, i.e. 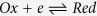). See more details on the preparation of this molecular layer junction in the experimental section and in the SI. Document (sections 1.1. and 1.2).

## Experimental Methods

Following previous works^22^,^25^,^26^,^27^, gold disk electrodes (2.0 mm diameter, METROHM) were mechanically polished with aluminum oxide pads or diamond spray on polishing cloth (Kemet) of progressively decreasing particle size: 1 μm, 0.3 μm and 0.05 μm, with intermittent sonication in water. The electrodes were then electrochemically polished in a deaerated NaOH or KOH 0.5 mol L^−1^ between the potentials −1.5 V and −0.5 V *versus* Ag|AgCl at a scan rate of 100 mV s^−1^ and then in deaerated 0.5 M H_2_SO_4_ between −0.2 V and 1.5 V at 100 mV s^−1^ until a stabilization of the gold reduction peak was noted (around 50 cycles). Electroactive areas were evaluated by integration of the cathodic peak from gold electropolishing voltammograms and converting to real surface area using a conversion factor of 400 μC cm^–2^. These determinations of area 0.033–0036 cm^2^ and the thickness (estimated herein as 1.45 – 1.45 nm) were used in the normalization of absolute recorded capacitance.

Electroactive Self-Assembled Monolayers (SAMs) were prepared by immersion of the polished gold electrodes (METROHM) in 11-ferrocenyl-undecanethiol (Sigma Aldrich) (1:100) for 16 h^22^,^25^,^26^,^27^. Accordingly prepared interfaces were then used for Electrochemical Capacitance Spectroscopy (ECS) measurements. For controlled temperature measurements a thermal bath was used in the range between 270 to 320 K with 30 min of delay time employed to stabilize temperature between each measurement.

All electrochemical measurements were undertaken on an AUTOLAB PGSTAT fitted with an FRA2 module. A three electrode cell setup was used with a gold (METROHM) working electrode, a platinum wire auxiliary electrode and a Ag|AgCl as reference electrode, providing a half wave potential of ferrocene molecule at about 0.45 V with respect to the reference, where electrochemical capacitive response is expected to be maximal^22^,^25^,^26^. Impedance spectra were collected between 1 MHz and 0.01 Hz with amplitude of 10 mV (peak to peak). All spectra were subsequently verified for compliance with linear systems theory by Kramers–Kronig by employing the FRA AUTOLAB software. The ECS analysis of these interfaces can be sensitively analysed measuring complex Z^*^(*ω*) (impedance) function and conversion into C^*^(*ω*). A more detail description of the experiments and ECS fundaments are given in the SI. document.

## Results and Discussions

**Figure 1:**
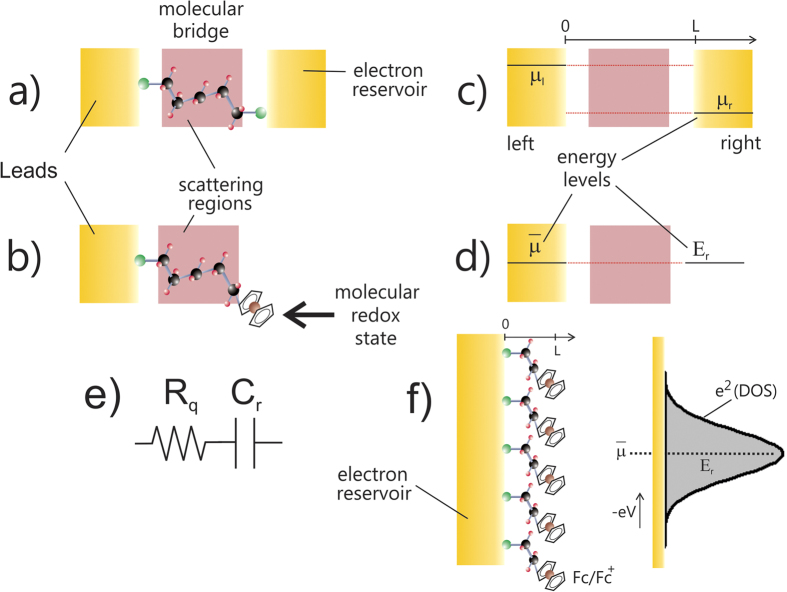
The propensity of molecules to transmit/charge can be probed experimentally by measuring charge-transfer rate constants (molecular electrochemistry) and molecular conductance (molecular electronics) in single or dual probe configurations, respectively. The electrodes can be considered as “leads” and the molecular dielectric/conductor as a “scattering region”. (**a**) In molecular conductance measurements, the molecular bridge links two electrodes (the latter acting as electron reservoirs or sinks), each containing a large density of states and the measurement is made in a non-equilibrium regime. (**b**) In charge transfer or molecular electrochemistry approaches, charge flows between discrete electronic states localized in the molecule to/from a high electrode density of states (and measurements are performed in a single probe experimental setup). Note that in (**b**) the transport and electron transfer processes are governed by *R*_*q*_ and *C*_*r*_ series parameters as shown in (**e**) within an outlined mesoscopic picture. In (**c**,**d**) the energy levels of the electrodes as measured in molecular conductance and molecular electrochemistry schemes, respectively, are depicted. The arrow in (**c**) depicts the direction of electron flux in DC measurements under an applied potential difference (chemical potentials of left and right electrodes depicted by *μ*_*l*_ on left and *μ*_*r*_ on right, respectively). The length of the scattering region is *L*. In the AC regime, (**d**), the conductive and capacitive characteristics are accessible either outside or at electrochemical equilibrium (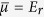), the latter obtained when the electron chemical potential of the electrons in the probe, 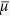, equates to that of molecular film, *E*_*r*_. Therefore, as depicted in (**f**), for a ferrocene redox pair (Fc/Fc^+^, i.e. reduced/oxidized) the electrochemical occupancy of these states is maximized and controlled by the density of states, DOS, i.e. 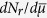, of the molecular layer that shapes as a Gaussian function shown in (**f**).

**Figure 2:**
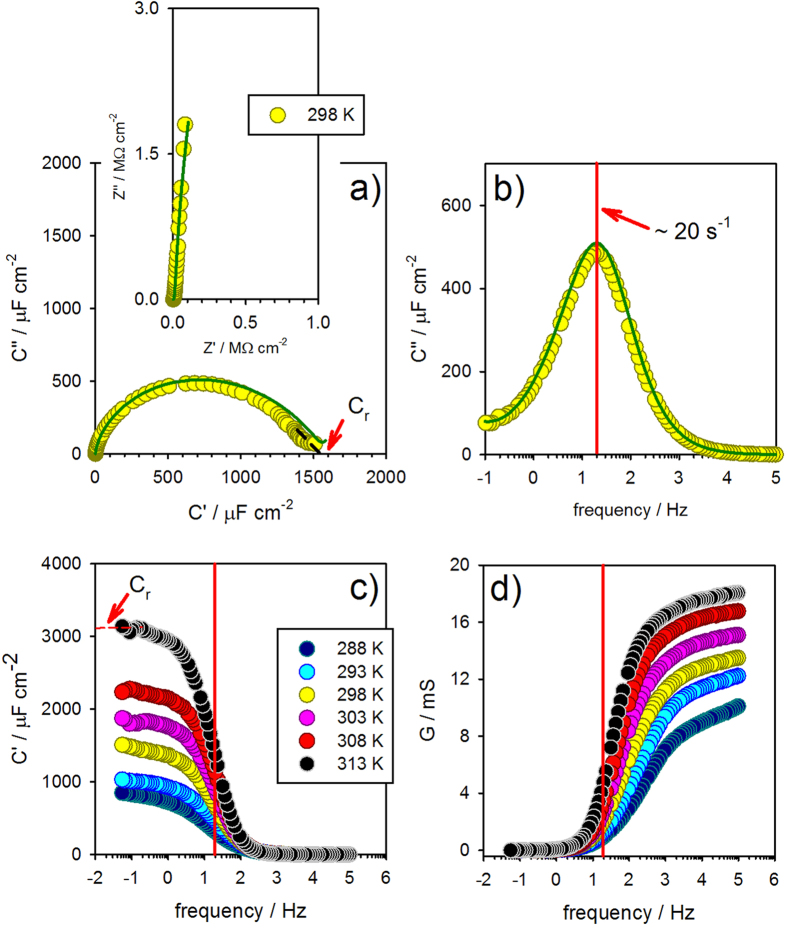
(**a**) A Nyquist representation of ECS for a 11-ferrocenyl-undecanethiol molecular film over an Au electrode indicating where the electrochemical capacitance, *C*_*r*_, can be obtained [alternatively also resolved as the low frequency plateau in (**c**)]. The inset Nyquist impedance plot resolves a vertical line with no real impedance component. The main capacitive plot confirms that capacitive effects are dominant. In (**a**,**b**) the green dark line corresponds to a fitting of a distribution of RC times (equivalent circuit model) to demonstrate the response is equivalent to a distribution of series resistances and capacitances; note that this fitting procedure is not necessary to obtain the RC parameters (which are accessible graphically). (**b**,**c**) are Bode representation of imaginary and real component of complex capacitance, respectively. Finally (**d**) shows the Bode diagram of AC conductance [*G*(*ω*) = *Y*′(*ω*) = *ωC*″], meaning the admittance of electrons to electrochemical/redox centres through the scattering region shown in Fig. 1b.

Within the context of a time-dependent (or frequency-dependent) analysis we have recently demonstrated that the charging signature of a molecular film junction containing accessible orbital states^14^,^22^ (Fig. 1b) is governed by the electrochemical capacitance (*C*_*r*_)^22^,^25^ and can be experimentally resolved by impedance derived capacitance analysis^27^,^28^ (see Fig. 2a and inset therein) and usefully applied within sensitive biosensor configurations^30^,^31^,^32^. The methodology enables a convenient mapping out of the redox density of states within the molecular layer and is termed Electrochemical Capacitance Spectroscopy (ECS)^27^. Importantly, as noted previously (above), it is a single probe measurement and, as such, requires no potentially perturbing or shorting secondary contact to be made to the molecules (constrained within the molecular film) of interest. Being an AC method, it intrinsically reports on dynamic (time-dependent) charge transfer/transport rather than that associated with a static DC imposed potential drop (see Fig. 1). In this time-dependent regime the electrochemical (or quantum) capacitance is associated with the phase shift between the imposed voltage oscillation and the observed current oscillation, with the quantum capacitance itself resolved at lower frequencies, where the imaginary component, C″, as exemplified by the end of the capacitive semicircle in Fig. 2a is null, and the real component of the complex capacitance function dictates the response at these lower frequencies region of the capacitive/conductive spectrum^22^,^25^,^27^,^28^. However, this implies that that conductance (*G* = *ωC*″) is null as shall be shown below and DC resistance is absent.

The quantum capacitance^22^, *C*_*q*_, of the film can be obtained as 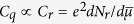, where 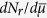 is the redox density of states and 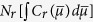 the density of redox states associated with accessible redox states in the film (exemplified in Fig. 1f with recently analysed ferrocenyl films ECS)^8^,^13^,^14^,^15^. This electrochemical capacitance (see also DFT analysis^14^) is intrinsically associated with the electron transfer/transport properties of the scattering region^25^. The primary purpose of the present work is to show specifically that the ECS approach can be powerful in resolving and quantifying the relaxation resistance (*R*_*q*_) associated with the dynamic charging of molecular redox states, reporting specifically on the electron transfer/admittance characteristics of the scattering region since the resonance frequency and *C*_*r*_ are known (see legend of Fig. 2). Once *R*_*q*_ has been defined (for instance, following the analysis of Fig. 2a,b) we can, in turn, report on the electron transfer rate and its correlation with relaxation conductance *G*_*q*_, inversely associated with resistance as 1/*R*_*q*_, as developed below. The analysis will be centred on the definition of a lifetime (*τ*) of the redox chemical process (see SI. 2.2) that relates *R*_*q*_ and *C*_*r*_ through *τ*_*r*_ = *R*_*q*_*C*_*r*_. This associates directly and proportionally with the phase shift of the electrochemical process and the scattering matrix^33^ for inelastic collisions.

Since the underlying principles of redox related storage and associated capacitance, *C*_*r*_, have been well described in previous work^22^,^25^,^26^,^27^, we herein focus initially on the scattering process that accompanies this electrochemical charging. The electron admittance (governed by the scattering region) associated with charging the addressable quantum states is given by Y^*^(*ω*) = [Z^*^(*ω*)]^−1^ = jωC^*^ (where asterisks denote a complex function and *ω* = 2*πf* states for the angular frequency and 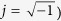 from which the AC conductance is obtained as *G*(*ω*) = *Y*′(*ω*) = *ωC*″ [where *Y*′(*ω*) is the real term of the complex admittance function and *C*″ the imaginary term of the complex capacitance function, calculated from electrochemical impedance measurements, see SI. document, section 1.2.]. Note that the structure of Fig. 1b exhibits no resolved DC transport features (as confirmed experimentally in the inset of Fig. 2a) but permits an AC (resonant) current flow (Fig. 2d) and an associated AC conductance. Ultimately it will be shown below that *G* is associated with the electron transfer rate, *k* = 1/*τ*_*r*_, obtained^22^,^25^,^26^,^27^, at the frequency of the peak (resonant frequency) observed in the imaginary capacitive Bode diagram (as indicated in Fig. 2b and having a value ∼20 Hz). The inverse of ∼20 Hz resonant frequency corresponds to the time scale of the redox process, i.e. *τ*_*r*_ = *R*_*q*_*C*_*r*_, from where the electrochemical conductance can be obtained as *G*_*q*_ = *C*_*r*_/*τ*_*r*_ = *kC*_*r*_ as shall be detailed below. To summarise thus far, ECS methods, when applied to an electrode configuration comprising a metallic probe and coupled redox sites, enable a facile resolution of electron admittance (AC conductance and thus electron scattering) in the molecular wire/bridge under an oscillatory electrical field.

As discussed previously, DC conductance (corresponding to large/infinite time and *ω* → 0) is not expected to be resolved at sufficiently low frequency when field-induced perturbations have enough time to relax to initial steady state conditions, leading to a pure capacitive response, as shown/confirmed in Fig. 2c. No information is obtained on the scattering region (the plateau of Fig. 2d), whereas capacitance is maximal and dominates (corresponding to the plateau of Fig. 2c), as expected for an equivalent circuit representation of the process [where *R*_*q*_ sits in series with *C*_*r*_ (see Fig. 1e)]. Both *C*_*r*_ and *R*_*q*_ are terms that contain, of course, mesoscopic information (i.e., terms that encapsulate molecular scale electronic characteristics) and thus information beyond that resolved by more traditional electrochemical analyses (e.g. empirically analysed and loosely termed pseudo-capacitance and charge transfer resistance). In exemplifying the quantum characteristics of the redox capacitance, we have recently demonstrated that this can be theoretically resolved into components as 1/*C*_*r*_ = 1/*C*_*e*_ + 1/*C*_*q*_, where *C*_*e*_ is the classical geometrical capacitance and *C*_*q*_ the quantum capacitance^22^,^29^. Important to note here is that the capacitance of the mesoscopic electrochemical structure ultimately depends, in an explicit manner, on the quantum mechanical density of states of the electrical conductor.

**Figure 3:**
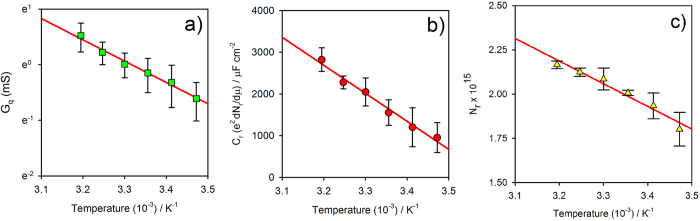
(**a**) The natural logarithm of the conductance (specifically the resonant admittance) of 11-ferrocenyl-undecanethiol redox molecular layer as a function of temperature. The linear dependence of the logarithm of *G*_*q*_ at the half-wave potential (where the electrochemical potential in the metal probe equals that of the molecular layer redox site) for a frequency of 20 Hz (the resonance frequency) with temperature. Such analyses resolve the reorganization energy of the redox process (about ~1.2 eV as reported in the text). (**b**) The values of *C*_*r*_ obtained at lower frequency [<0.1 Hz, corresponding to any region of the plateau indicated in Fig. 2c] at the half-wave potential (or at the electrochemical equilibrium potential as discussed in the text) is shown to be proportional to *G* with an equivalent thermal dependence as expected according to 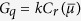. (**c**) The integrated value of the redox DOS in (**a**) directly provides *N*_*r*_ and its dependence with temperature that, as expected, follows that of *G* obtained in Fig. 3a. All plots are obtained from averages over at least three different molecular films.

The temperature dependence of electron transport/transfer across the scattering region is, of course, useful in providing additional information about the operating physical mechanisms of redox reaction processes. Electrochemistry is essentially a thermally activated process and we show herein that the resolved (Fig. 2c,d) thermal activation correlates (see below) with Marcus’s (electron transfer rate) theory^34^,^35^ built on transition state theory^4^ (see legend of Fig. 3 for more details), as expected.

Therefore, within the framework of considering the redox site charging to be mesoscopic in character, we consider electron transport within a wave-packet^33^ (or wave-train) analysis (see S.I. 2.2.). By using the latter formalism we ensure that the integer electron transfer character associated with the electrochemistry is retained but, at the same time, the wave nature of the electron during the electrochemical reaction is considered. In the zero temperature limit (although such a limit allows Fermi energy terminology to be used we prefer to retain the term Fermi level, 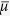, to avoid adopting an additional nomenclature for zero temperature limit) the transfer rate of electronic packet, is thus associated with a lifetime *τ* = 1/*k* (the time scale of the associated electrochemical event) where 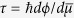 and *ϕ* is the phase shift due to the scattering region between metallic and redox states in the molecular film (see S.I. 2.2). The term 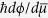 comes from Wigner-Smith time-delay matrix that defines^33^ a lifetime (and a lifetime matrix) as the duration of an involved scattering event plus the residence in the quantum electrochemical states. In other words, the difference (Δ*t* = *τ*) between the time associated with the perturbed electron entering and leaving the scattering region equates to the traversing time plus the time the electron is resident on the redox site. This, in turn, is inversely proportional to the electron transfer rate, *τ*_*r*_ = 1/*k*. Accordingly, *τ*_*r*_ is associated with the scattering matrix for inelastic collisions as 
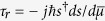
, where *s* is the scattering matrix, 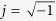 and *ħ* = *h*/2*π*. Considering the equilibrium (low frequency) density of states 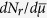 (corresponding to the capacitance 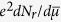 accessible experimentally from the lower frequency plateau in Fig. 2c), i.e. after the perturbed and transferred charge to the electrochemical/redox states relax back up to its original stationary state, *s* equates to *e*^*jϕ*^**, where *ϕ* is the phase a charge accumulates from its entrance into the quantum channel up to its reflection (essentially translated into a transient occupancy) in the quantum electrochemical states and finally exit back to the reservoir/electrode.

For multiple channels 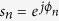 with *n* denoting the number of eigen channels at a given Fermi level, 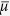, and *ϕ*_*n*_ is thus the total phase a charge/carrier accumulates from its entrance into the *n* state up to its exit back to the electron reservoir. Now by assigning one channel as being available at each molecular site, 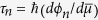 , is the average time associated with electrochemical transient frequency. Essentially, therefore, the conductance *G* can be expressed in terms of *τ*_*n*_ [see S.I. document and Eqn. (10) stated in reference^36^ for more details], in the zero temperature limit, as^29^


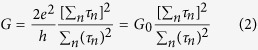


Note that *G* (which is herein presented as the inverse of *R*_*q*_ – see more details in SI. document and references indicated therein) is essentially determined from (or proportional to) the square of the sum of the electrochemical lifetimes divided by the sum of the squares of these lifetimes. Note either that the quantized quantity of charge relaxation resistance (*h*/2*e*^2^) as discussed by Buttiker^36^ (see Eqn. 10 therein) is equivalent to the quantum of the conductance (*G*_0_ = 2*e*^2^/*h*) if inverted, i.e. the inverse of the resistance is the conductance. This resistance unit is not the von Klitzing *h*/*e*^2^ but instead *h*/2*e*^2^, i.e. a half of it. The factor two arises since the quantum state is coupled to one reservoir only and thus only the half of energy is dissipated. These observations are consistent with the electrical conductance of a quantum conductor being twice the reciprocal of the von Klitzing constant (*h*/*e*^2^).

Note also that Eqn. (2) can be derived by considering that the electrochemical density of states is 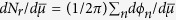 and so that the capacitance can be written as


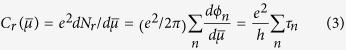


**Figure 4:**
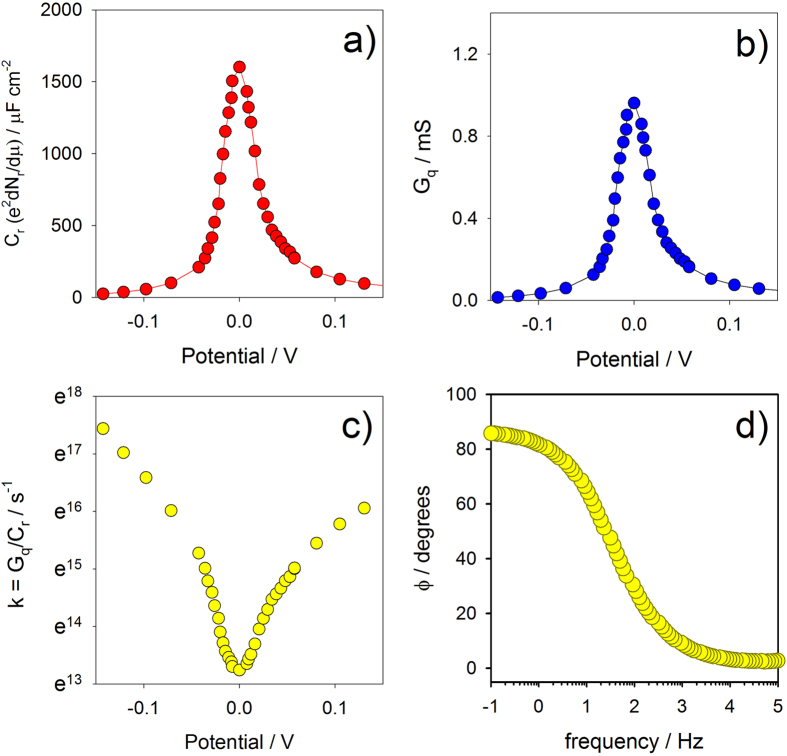
(**a**) Redox capacitance, *C*_*r*_, as a function of potential (energy) reflecting the redox density of states 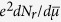 for a 11-ferrocenyl-undecanethiol redox molecular layer at 0.1 Hz (see data in SI. document section 1.2). (**b**) The molecular film conductance (calculated by taking 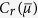 at frequencies lower than 0.1 Hz and the resonant frequency at 20 Hz) as a function of potential. Both *C*_*r*_ and *G*_*q*_ maximize when electrode energy is aligned with redox site, *E*_*r*_ [according to Eqn. (6)]. (**c**) Rate constant 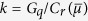 is in agreement with the Marcus’ theory predicted behaviour at electrode^39^ as a function of potential [more details on how these curves were obtained are given in the SI. document]. In (**a**–**c**) note that data was obtained at room temperature (298 K) and at the equilibrium electrochemical potential, i.e. 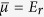 and was here zeroed just for convenience. (**d**) depicts the phase, *ϕ*, as a function of frequency at room temperature and at 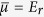. Indeed the phase follows *C*_*r*_ as predicted by Eqn. (3).

Eqn. (3) was obtained by noting that 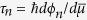 is the time the charge/carrier in the *n*-th mode of each electrochemical channel spends in the molecular layer (within the scattering region plus the time spent on the molecular redox film) before being scattered back to the reservoir/electrode. Eqn. (3) predicts that 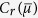 is proportional to the phase shift, an observation which is indeed experimentally observed (Fig. 4d). The obtained/associated averaged electrochemical dwell time is given by 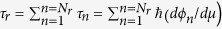, where *N*_*r*_ is the density of electrochemical states, experimentally accessible by ECS at finite temperature (see Fig. 4a). Accordingly 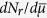 is proportional to the number of electrochemical states at 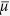 ultimately proportional to the average dwell time *N*_*r*_*τ*_*r*_/*h*. In assuming a single energy state model (meaning simply treating all redox sites as identical) and either only a single RC channel thus *N*_*r*_ = 1 and Eqn. (2) is universal, leading to the quantized conductance *G*_0_, as expected.

Experiments are accessible at room temperature in which 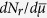 is a continuum function (the Gaussian function experimentally obtained as shown Fig. 4a) and, therefore, for the finite temperature limit, Eqn. (2) turns into


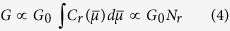


From where it can be confirmed that *G* is directly associated with the density of electrochemical states (*N*_*r*_) communicating with the electrode states obtained by integrating 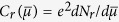 function, i.e. the Gaussian function of Fig. 4a, over all the energy states (or over a given energy range of interest). Eqn. (4) implies that the bulk conductance of a redox interface/film increases proportionally with an increase in the density of electrochemical states accessible from the electrode states because each of them brings an associated quantum/mesoscopic channel (and dwell time) connecting to metallic probe states. Therefore, in the experimentally realistic multiple channel limit, 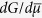 is proportional to 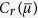 and *G* to *N*_*r*_. Finally note also that, in assuming a single energy state model, *N*_*r*_ equates to molecular coverage Γ (see also section 1.2 of the SI. document)^22^,^25^.

We can note as *G*_*q*_ (at the resonant frequency) and *C*_*r*_ (at the low frequency plateau as indicated in Fig. 2c) are both proportionally connected through *τ*_*r*_ by the relationship 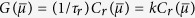 that can be used to plot functions as those shown in Fig. 4. Through the complex capacitance function (obtained from ECS measurements^14^,^22^,^28^) it is possible to establish the correlation between *C*_*r*_ and *τ*_*r*_ (by considering that *R*_*q*_ and *C*_*r*_ couples in series following the Kirchhoff’s rules) and its time-dependent response according to


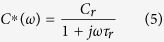


where it can be noted that for long times, *t*, (*ω* → 0), i.e. *t* ≫ *τ*_*r*_, *C*_*r*_ dominates and the *G* (approaches the DC conductance at infinite time from where transmittance is zeroed) is unresolved tending to negligible (null) values as expected for DC limit (low frequency). On the other hand, an inverse behaviour is observed for (*ω* → ∞), i.e. *t* ≪ *τ*_*r*_, where *C*_*r*_ is zeroed since only transmittance are allowed and none scattering of electrons back to the reservoir are accessible (there is not enough time for electron relaxation to occur). Experimentally, and as noted above, this complementary behaviour between *G* and *C*_*r*_ is resolved by the ECS measurements depicted in Fig. 2c,d; at frequencies lower than 1 Hz the capacitive behaviour predominates meaning, as explained previously, that the interface is in a stationary DC regime where perturbed electrons have moved and then had sufficient time to relax to initial states. At this DC regime *G* is unresolved/zeroed as expected theoretically and the response is purely capacitive. The inverse is observed at higher (>1 kHz) frequencies where *G* predominates over a capacitive behaviour that is now itself unresolved/zeroed (following the expected behaviour of the complex capacitance function stated in Eqn. 5). One must be aware that neither the resistance nor capacitance circuit elements here is, in reality, ideal specially in the sense that they are not classical but contain quantum information. Therefore, any associated classical equivalent circuit model is not able to reproduce observations related to the presence of series quantum mechanical terms *R*_*q*_ and *C*_*q*_. Herein we see that the classical series RC circuit traces the pattern shown in Fig. 2a,b, but not the observed depression evident in the Nyquist diagram of Fig. 2a. This non-ideality arises from a deviation from a Debye ideal relaxation phenomenology (those relaxation related with the time scale associated with a series resistance and capacitive terms, where a single RC relaxation time exist) that needs to consider the Gaussian shape of *C*_*q*_, derived from capacitance, in any subsequent fitting. This is precisely because of the thermal distribution of quantized redox state energies relevant to any realistic electrochemical system. The fittings shown in Fig. 2a,b demonstrate that this improved consideration of energetic dispersion does indeed track the non-ideal Nyquist shape experimentally observed. Note that this behavior is associated with the existence of a non-Nernstian behavior (due to the quantized character of the states) of these molecular monolayers since the Nernstian statistics follow a Boltzmann phenomenology, i.e. purely classical statistical mechanic instead of more convenient quantum statistical mechanics for electrons in the redox molecular layer^26^. The observed depression of the semi-circle is thus associated with how the redox molecular states disperse with the dielectric of the electrode environment as we have previously observed^22^,^25^.

For finite temperatures the previous outlined theoretical treatment of electron transfer/transport within electroactive molecular layers connects neatly with Marcus’s theory^37^; derived from transition state theory and Fermi’s golden rule, 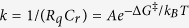, where Δ*G*^‡^ is the standard activation free energy, *T* is the absolute temperature, *k*_*B*_ is the Boltzmann constant and *A* the pre-exponential factor that encompasses electronic coupling between the electronic states (including Fermi-Dirac distribution, see SI. document section 2.3) and those in redox molecular layer. Key within this treatment as applied to electrode confined redox sites is the relationship between the Gibbs free energy of the electron transfer and the imposed electrode driving force 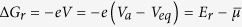, between metallic and molecular states^4^. Δ*G*^‡^ can thus be written as 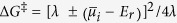, where 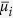 is the electrochemical potential of electrons in the *i*-th metallic state and *λ* the well-known nuclear reorganization energy of the electron transfer process. As the potential of the electrode is potentiostatically modulated, the alignment between 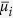 (metallic states) and those of molecular layer (*E*_*r*_) can be readily tuned in a standard electrochemical measurement^4^. Thus taking the situation where 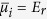 than Δ*G*_*r*_ is null (Δ*G*^‡^ = *λ*/4) and *G*_*q*_ (at the resonant frequency) is given by


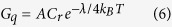


From Eqn. (6) *λ* can be obtained experimentally from ECS data by noting the expected linear behaviour of ln(*G*_*q*_) (with *G*_*q*_ obtained at the resonance frequency of 20 Hz of Fig. 2d, i.e. 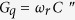) *versus* 1/*T* as shown in Fig. 3a. Since Eqn. (6) applies for 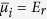 (i.e. when the electron chemical potential of electrode is aligned with that of molecular layer – this is achieved by poising the potential of the electrode at the half-wave or formal potential), the resolved *G*_*q*_ is that corresponding to an energetic alignment between the Fermi level in the metal and those of the electrode-confined quantum states. The obtained value of *λ* resolved here is ~1.2 ± 0.15 eV in fair agreement within theoretical predictions (0.94 eV)^38^ and experimental observations by others^39^ (for films of this type as analysed using different methodologies). The solvent reorganization energy is calculated from the dielectric continnum model (as stated by Marcus) for ferrocene with a 3.8 angstrons solvation radius in water 20 angstrons from a metallic electrode. The theoretical calculated value assumes that the ion is bathered in an infinite bath of water, whereas the real ion is flanked by a 20 angstrons-thick sheet of hydrocarbon. A double check of this resolved *λ* by calculating *G*_*q*_ as 1/*R*_*q*_ = *C*_*r*_*k*_*r*_ obtained graphically from Fig. 2a (i.e. by the Nyquist plot where *C*_*r*_ and *k*_*r*_) and Fig. 2b (where *k*_*r*_ is obtained as the frequency value of the peak) reports *λ* as ~1.0 ± 0.2 eV (the obtained results for *λ* are in agreement within the experimental errors associated with each method).

In Fig. 4a,b we observe the density of state functions constructed for *C*_*r*_ and *G*_*q*_, respectively, at energies around *E*_*r*_ (see SI. document sections 1.1 and 1.2 to access information on how these curves were constructed). In combining the 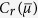 function obtained at low frequency (in the plateau of Fig. 2c) with the conductance obtained at the resonant frequency as 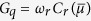 (where *ω*_*r*_ = 2*πf*_*r*_ and *f*_*r*_ ~ 20 Hz) it is possible to generate a third function, i.e. 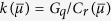 as shown in Fig. 4c. The integrated value of *C*_*r*_ along all the energy states supports a calculation of *N*_*r*_ and consequently its dependence with temperature (Fig. 3c). As expected, this follows that of *C*_*r*_ (Fig. 3b) due to the expected proportionality. The ratio 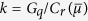 (Fig. 4c) as a function of potential provides the expected behaviour of electrode-confined Marcus’s theory^37^,^39^ where *k*_*r*_ is minimal around *E*_*r*_ (set in Fig. 4c as null for convenience) and tends to a plateau at higher driving force^39^. This analysis is, of course, equivalent to the classical Tafel’s plot^4^ trends empirically/phenomenological followed by Eqn. (1). Indeed, following previous work^25^,^26^, for single redox energy state, the equilibrium occupancy between electrochemical and metallic states (governed by Fermi-Dirac distribution, 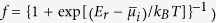 was shown to be *C*_*r*_ = (*e*^2^*N*_*r*_/*k*_*B*_*T*)*f*(1 − *f*)^25^ (see SI. document for more details). Thus Eqn. (6) can be generically written as


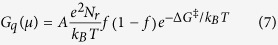


with a maximum of 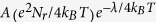 predicted (and confirmed in Fig. 4b) when *f* = 1/2, i.e. at the half-wave potential which poises the electrode at an energy corresponding to the Fermi level of the molecular states *E*_*r*_ (where Δ*G*^‡^ = *λ*/4). The shape *G*_*q*_(*μ*) as a function of electrode potential driven by 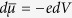 is controlled by the shape of 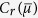 in a manner dependent on the solvent environment^25^, i.e. the rate constant *k* is predictably affected by the dielectric of the environment (see Fig. 3 of reference^22^). This trend in turn will be responsible for conductance dispersion as a function of solvent dielectric, a subject of on-going work.

The relationship between *G*_*q*_ and *k* noted previously^13^,^40^ is analysed with some difficulty in DC experiments (involving donor-bridge-acceptor systems, for instance). The challenge arises largely through an acknowledgement that the resolved electronic structure of “the molecule” is influenced by the process of integrating it with the leads, particularly the “top lead”. Within this non-equilibrium DC experimental arrangement (Fig. 1a,c) the density of states associated with the capacitive coupling is not experimentally accessible, making the analyses very demanding. Despite these complications, DC analyses have been extensive^2^,^3^,^5^,^6^,^19^,^20^,^21^ and, have indeed shown^13^ that these two charge transport (conductance) and electron transfer rate processes are related by a power law^13^ (although this relationship has not been understood). In utilising the *natively single contact* ECS approach, *k* and *C*_*r*_ are experimentally accessible to resolve *G*_*q*_. Indeed we have shown here that *G*_*q*_ term is obtained by accessing the redox density of states contained in *C*_*r*_ and by additionally resolving *k*. Furthermore, the density of electrochemical states, *N*_*r*_, and *G*_*q*_ can be obtained through *C*_*r*_ and the electrochemical delay time (accessible through the resonant frequency) for any molecular component acting as a bridge between metallic and redox states through AC measurements. This we believe to be true with an unprecedented experimental facility.

## Conclusions and Final Remarks

In summary, the predictable alignment between conductance and electron transfer rate previously explored both theoretically^40^ and experimentally^13^ through a variety of methods (all DC in essence) was herein theoretically underpinned from a quantum mechanical perspective and resolved by single probe time-dependent ECS experiments. Within this we have proposed a mesoscopic approach for electrochemistry in which electrochemical processes can be understood in terms of a mesoscopic conductance 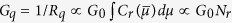 and its relationship with *C*_*r*_ through the electron transfer rate, *k*. The latter represents the inverse of the lifetime (given by the individual quantum states 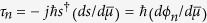 associated with each electrochemical states) related to the electrochemical relaxation (consequence of a voltage fluctuation) of the redox reaction and in turn is connected with the phase shift of the electron transfer/transport associated with charging the redox quantum states. Furthermore, the alignment between this approach and Marcus’s semi-classical (electron transfer rate) theory enables reorganisation energy to be resolved^4^,^37^,^39^. Ultimately, the model proposed is based on both the scattering (*G*_*q*_ = 1/*R*_*q*_) and storage (*C*_*r*_) mesoscopic terms which replace those empirically known as charge transfer resistance^4^ and pseudo-capacitance^41^, respectively.

## Additional Information

**How to cite this article**: Bueno, P. R. *et al*. The Mesoscopic Electrochemistry of Molecular Junctions. *Sci. Rep*. **6**, 18400; doi: 10.1038/srep18400 (2016).

## Supplementary Material

Supplementary Information
