# Supplementary material for: The Mesoscopic Electrochemistry of Molecular Junctions
Source: Sci Rep. 2016 Jan 13;6:18400. doi: 10.1038/srep18400 (PMC4725828; doi:10.1038/srep18400)
Supplement: Supplementary Information [file srep18400-s1.pdf]

SUPPORT INFORMATION

Mesoscopic Electrochemistry of Molecular Junctions

Paulo R. Bueno[1]*, *Tiago Azevedo Benites*[1], *Jason J. Davis*[2]

[1]Institute of Chemistry, Physical Chemistry Department, Univ. Estadual Paulista (São Paulo State University, UNESP), Nanobionics group (www.nanobionics.pro.br), CP 355, 14800-900, Araraquara, São Paulo, Brazil.

[2]Department of Chemistry, University of Oxford, South Parks Road, Oxford OX1 3QZ, UK.

SI. 1. EXPERIMENTAL PROCEDURES

SI. 1.1. Molecular layer over electrodes

Following previous works[1-4], gold disk electrodes (2.0 mm diameter, METROHM) were mechanically polished with aluminum oxide pads or diamond spray on polishing cloth (Kemet) of progressively decreasing particle size: 1 µm, 0.3 µm and 0.05 µm, with intermittent sonication in water. The electrodes were then electrochemically polished in a deaerated NaOH or KOH 0.5 mol L^{-1} between the potentials -1.5 V and -0.5 V *versus* Ag|AgCl at a scan rate of 100 mV s^{-1} and then in deaerated 0.5 M H_2SO_4 between -0.2 V and 1.5 V at 100 mV s^{-1} until stabilization of the gold reduction peak (around 50 cycles). Electroactive areas were evaluated by integration of the cathodic peak from gold electropolishing voltammograms and converted to the real surface area using a conversion factor of 400 µC cm^{-2}. These determinations of area 0.033-0036 cm^2 and the thickness (estimated herein as 0.80-085 nm) were used in the normalization of absolute recorded capacitance.

Electro active Self-Assembled Monolayers (SAMs) were prepared by immersion of the polished gold electrodes (METROHM) in 11-ferrocenyl-undecanethiol (Sigma Aldrich) (1:100).[1-4] Electrodes prepared according to the above procedure were finally used as working electrode for Electrochemical Capacitance Spectroscopy (ECS) measurements. For controlled temperature measurements a thermal bath was used to control/change temperatures in the range between 270 to 320 K with 30 min of delay time employed to stabilize temperature between each measurement. The electrochemical reaction under analysis here concerns the redox pair given by [AuS-$(CH_2)_{11}$-Fc]/[AuS-$(CH_2)_{11}$-Fc^+] where only the ferrocene group shows accessible redox chemistry.

SI. 1.2. Electrochemical Capacitance Spectroscopy Measurements and Summarized Theory

All electrochemical measurements were undertaken on an AUTOLAB PGSTAT fitted with an FRA2 module. A three electrode cell setup was used with a gold (METROHM) working electrode, a platinum wire auxiliary electrode and a Ag|AgCl as reference electrode, providing a half wave potential of ferrocene molecule at about 0.45 V with respect to the reference, where electrochemical capacitive response is expected to maximize.[2-4] Impedance spectra were collected between 1 MHz and 0.01 Hz with amplitude of 10 mV (peak to peak). All the impedance spectra were subsequently verified for compliance with linear systems theory by Kramers–Kronig by employing the FRA AUTOLAB software. The ECS analysis of these interfaces can be sensitively analysed measuring complex $Z^*(\omega)$ (impedance) function and conversion into $C^*(\omega)$ (capacitance) by $C^*(\omega) = 1/i\omega Z^*(\omega)$, where ω is the angular frequency and $i = \sqrt{-1}$ (i.e., complex number). Herein we then indicate that an asterisk refers to complex conjugate functions, for instance, $C^*(\omega) = C' + C''i$ where C' and C'' are, respectively, the real and imaginary parts of the complex capacitance function. Therefore, practically ECS procedures involves taking the data resolved in a standard impedance analysis ($Z^*(\omega)$), sampled across a range of frequencies at any steady-state potential, and converting it phasorially into complex capacitance [$C^*(\omega)$] with its real and imaginary components. In processing $Z^*(\omega)$ datasets in this way one obtains the imaginary part of the capacitance by noting that $C'' = \varphi Z'$ and real part as $C' = \varphi Z''$, where $\varphi = (\omega|Z|^2)^{-1}$ and $|Z| = \sqrt{(Z'')^2 + (Z')^2}$ is the modulus of Z^*. If one carries out this analysis inside of the surface potential window where redox activity is observed and then outside the potential window, the difference (between the two impedance measurements) is obtained as the pure electrochemical response constituting of a series resistance and capacitance[1,4], as fitted in Figure 2*a* and 2*b*. It should be noted that, in the absence of a redox film, charging capacitance is comparatively very small for higher molecular coverage and C_r can, alternatively, be estimated by a simple subtraction of the former.[1,4] Plotted data points (in Figure 3) represent mean and standard deviations values across (at least) three different measured junctions (molecular film/electrode).

In more detail Electrochemical Capacitance Spectroscopy (ECS)[1] is a methodology based on the resolved ratio between a time-dependent current response and an imposed small amplitude voltage applied to molecular scale films. We have previously shown that this enables a quantification of an electron chemical (redox) capacitance and its associated quantum components within energetically addressable molecular films,[2,3,5] and that these charging characteristics, in the past loosely ascribed to “pseudo capacitance” (or faradaic capacitance),[5] represent a specific manifestation of the general mesoscopic capacitance[6] principles introduced by Marcus Buttiker. This electron chemical capacitance, is redox (faradaic) capacitance (C_r) where energetic donor/acceptor states are associated with reduced and oxidized chemical states in the molecular film, arises specifically from the coupling of a supporting electrode density of states [$g_m(\bar{\mu})$] to the redox sites molecular density of states [$g_r(\bar{\mu}) = dN_r/d\bar{\mu}$], where $\bar{\mu}$ is the electron chemical potential of the electrode, constituting an energy $d\bar{\mu} = -edV$, where V is the electrostatic potential of the electrode (applied with respect to the equilibrium potential – see main text) and e is the elementary charge. We note/demonstrated previously that this relates to the concepts introduced by Serge Luryi[7] in his consideration of quantum capacitance in devices comprising an interphase boundary of metal with a two-

dimensional electron gas system. It is also resolved free of non-faradaic capacitive components, i.e. those not associated with redox state occupancy[2,5]. We have shown that C_r is comprised of two distinct series contributions thus $1/C_r = 1/C_e + 1/C_q$, where C_e is the electrostatic or geometrical capacitance arising from charge separation in the normal (classical) sense and C_q the quantum capacitance, arising very specifically from the chemical potential changes associated when charging a nanoscale (atomic/molecular scale) entity.[2,5]

As the electrochemical capacitance C_r, as an additional chemical interfacial charging, can be spectrally resolved, as demonstrated previously here, its applications into molecular films are useful in sensorial applications and devices[8-10]. Since this interfacial charging depends on the quantum mechanical coupling of the electrochemical states and electrode/metallic states, the former spanning a Gaussian in distribution (at finite temperature), one can quantify by integrating over all contributing energy levels thus

$$C_r(\bar{\mu}) = e^2 \int_{-\infty}^{\infty} g_r(\bar{\mu}) \frac{df}{d\bar{\mu}} d\bar{\mu} = \frac{e^2}{k_B T} \int_{-\infty}^{\infty} g_r(\bar{\mu}) f(1-f) d\bar{\mu} \qquad \text{(SI. 1)}$$

where, $g_r(\mu)$, the redox density of states can be written as

$$g_r(\bar{\mu}) = \frac{dN_r}{d\bar{\mu}} = \frac{1}{\sigma_g \sqrt{2\pi}} \exp\left[-\frac{(\bar{\mu}-E_r)^2}{2{\sigma_g}^2}\right] \qquad \text{(SI. 2)}$$

i.e., a Gaussian function with the normal distribution centred at E_r (the energy of the redox states) and with a σ_g standard deviation (${\sigma_g}^2$ variance). Any change in redox site occupancy is thus integrated into $g_r(\bar{\mu})$ and experimentally reported through C_r. Note that f is the Fermi-Dirac distribution $f = \{1 + \exp[(\bar{\mu} - E_r)/k_B T]\}^{-1}$ and that at zero-temperature limit SI. Eqn. (1) turns into $C_r(\bar{\mu}) = e^2 g_r(\bar{\mu}) = e^2 dN_r/d\bar{\mu}$. The N_r (the density of redox states) is then occupied according to electrochemical chemical potential of the electrons in the electrode following the Gaussian DOS shape where k_B is the Boltzmann constant and T the absolute temperature. Note also that $\bar{\mu}$ is related to the Gibbs free energy of a single electron transfer step according to $\Delta G_r = E_r - \bar{\mu}$, where the electrical work defined by edV translates into associated with change in free energy and ultimately stored chemical energy, i.e. $d\bar{\mu}$.

Table SI. 1. shows the data of conductance from where Figure 3*a* was obtained and Table SI. 2. explains how Figures 4*a* to 4*c* were generated and tabulates the data of Figure 4*a*.

Table. SI.1. Tabulated data of conductance (G values) obtained at the half-wave potential (i.e. equivalent in poising the electrode at the electrochemical potential of the molecular layer where thus $\bar{\mu} = E_r$) as $G = \omega C''$ from Figure 2 for a frequency of 20 Hz. This data was used to construct Figure 3*a*.

Temperature (K)	Conductance (mS)
288	1.35
293	1.80
298	2.14
303	2.50
308	3.10
313	4.18

Table. SI.2. Tabulated data of Figure 4*a* of $C_r(\bar{\mu})$ from where G_q was obtained by taking the resonant frequency of 20 Hz. Figure 4*b* was then calculated as $G_q = (2\pi\ 20\ \text{Hz})\ C_r(\bar{\mu})$. The behavior of the natural logarithm of the electron transfer rate as a function of potential is shown in Figure 4*c* and was calculated by observing that $k(\bar{\mu}) = G_q/C_r(\bar{\mu})$ at 20 Hz.

Potential / V	C_r / μF cm^{-2}
-0.1425	2.40E-05
-0.1212	3.65E-05
-0.0976	5.63E-05
-0.0715	1.00E-04
-0.0426	2.10E-04
-0.036	2.73E-04
-0.033	3.40E-04
-0.0287	4.14E-04
-0.026	5.23E-04
-0.0219	6.50E-04
-0.0201	8.26E-04
-0.0171	9.96E-04
-0.0148	1.15E-03
-0.0112	1.28E-03
-8.29E-03	1.39E-03
-7.47E-03	1.51E-03
5.20E-18	1.60E-03
7.71E-03	1.43E-03
9.41E-03	1.32E-03
0.0119	1.22E-03
0.0161	1.02E-03
0.0205	7.85E-04
0.0249	6.52E-04
0.0294	5.58E-04
0.034	4.69E-04
0.0385	4.26E-04
0.0433	3.86E-04
0.048	3.39E-04
0.0528	3.16E-04
0.0575	2.74E-04
0.0575	2.72E-04
0.0805	1.76E-04
0.1048	1.26E-04
0.1305	9.59E-05
0.1576	7.75E-05

SI. 2. THEORETICAL BACKGROUND

SI. 2.1. Quantum Transport (in time-independent regime)

The physics associated with molecular scale electronic[11] remains high profile within the scientific community since it impacts on both developing theoretical treatment and the successful fabrication of nanoscale devices. The modelling of such devices is complicated from theoretical point of view involving the self-consistent solution of the Schrödinger equation. The majority of problems are focused on transport in non-equilibrium condition as exemplified in Figure 1*a* of the main text. Therefore, the experimentally studied devices contain narrow quasi-one-dimensional obstructions to conduction and so that the conductance of such systems is generally quantised. The electrons in such quasi-one-dimensional conductor occupy a set of sub-bands (or quantum wells) due to a quantisation of the electronic states in the transverse direction of the quantum wells. In assuming there is no scattering of electrons between sub-bands or quantized wells, the electrons in each sub-band (or quantized energies) can be considered as a one-dimension electron gas. Therefore, in a one-dimensional DC conductive regime the density of states at energy, $\bar{\mu}$, for electrons propagating in one direction is

$$\frac{dN}{d\bar{\mu}} = 2(L/2\pi)\frac{dk}{d\bar{\mu}} \qquad \text{(SI. 1)}$$

where L is the length of the conductor and k is the wavenumber of the electronic states. The group velocity of the electrons that have energy E is

$$v(\bar{\mu}) = (1/\hbar)\frac{d\mu}{d\bar{k}} \qquad \text{(SI. 2)}$$

In considering that all the electronic states propagating to the right in the energy range between $\bar{\mu}$ and $\bar{\mu} + \Delta\bar{\mu}$ are occupied and all states propagating to the left are unoccupied the current carried by the electrons in the energy ranges of $\Delta\bar{\mu}$ is given by

$$I = (e/L)\frac{dN}{d\bar{\mu}}v(\bar{\mu})\Delta\bar{\mu} = (2e/h)\Delta\bar{\mu} \qquad \text{(SI. 3)}$$

which is independent of $\bar{\mu}$ and now considering that the energies over which all the right-propagating (from lower to high potential in Figure 1*a* and Figure 1*c*) electronic states are occupied and the left-propagating states are unoccupied were equal to eV, where V is the potential (or voltage difference) across the conductor (see Figure 1*a* and Figure 1*c*), the conductance of the system would be

$$G = \frac{I}{V} = \frac{2e^2}{h} \qquad \text{(SI. 4)}$$

Finally, Eqn. (SI. 4) suggests that each occupied sub-band in a quasi-one-dimensional conductor should have a quantised conductance[12], in which case the conductance of the devices studied experimentally would be

$$G = \frac{2e^2}{h} n \quad \text{(SI. 5)}$$

where n is the number of occupied sub-bands (quantum wells) in the narrow constriction. Although Eqn. (SI. 5) is able to describe some particular experiments, there are a lot of difficulties with SI. 5 including the fact that electrons move from a wide region of the device (the electrode or electron reservoir) into the quasi-one-dimensional constriction. Eqn. (SI. 5) is then not able to predict the transverse width of the device, etc. Applications of the Landauer multi-channel conductance formula[12,13] leads to

$$G = \frac{2e^2}{h} T_r\left(t^\dagger t\right) \quad \text{(SI. 6)}$$

where t is the transmission matrix. Eqn. (SI. 6) predicts the quantum conductance for a quasi-one dimensional conductor if the magnitude of the transmission coefficient for each is unity. In the simplest case where the scattering matrix of the conductors does not depend on the energy Eqn. (SI. 6) reads $G(\bar{\mu}) = G_0 \sum_n T_n\,(\bar{\mu})$, where $G_0 = 2e^2/h$ is the quantum of conductance ($\sim$7.75 x 10^{-5} Ω^{-1}) and T_n are the transmission eigenvalues of the channels. If the sub-bands are continuum (meaning the spectrum of electrons are continuum) the sum runs over all the transport channels in the conductor at all possible energies and so that the conductance is given by $G(\bar{\mu}) = G_0 \int T\,(\bar{\mu}) d\bar{\mu}$. Therefore, the conductance of a nanoscale conductor is given by the sum of all the transmission probabilities an electron has when propagating with an energy equal to the electron chemical potential, i.e. $E = -eV = \bar{\mu}$.[12,14]

The problem with Eqn. (SI. 6) is that it does not take into account self-consistency in a way that a more appropriate formulation of the problem that take into account the self-consistency was proposed by Buttiker[13] and today is known as Landauer-Buttiker formalism

$$G = \frac{2e^2}{\pi\hbar} T_r\left(\sum_i T_i\right)\left(\sum_i v_i^{-1}\right)/\left[\sum_i v_i^{-1}\,(1 + R_i - T_i)\right] \quad \text{(SI. 7)}$$

where the sum is over all incident channels and v_i, R_i and T_i are the longitudinal velocity, reflection coefficient and transmission coefficient for channel i.

SI. 2.2. Lifetime in Collision Theory and Electrochemistry

The time-dependent analysis of the capacitance in redox molecular films in the present work and in other from us[1-4] has been focused on the mesoscopic characteristics of organic films. Herein specifically we emphasize the use of time-dependent transport to gain information on the charge transfer and transport characteristics of the redox film and its collective (ensemble) dynamics, i.e. multiple redox states connected to or resonating with metallic states of the electrode (which implies conformational as well). It means the treatment is not concerned with stationary time-independent regime (as shown in Figure 1*a*) of the atomic or molecular structures involved, but in driving them from one state to another (for instance, in linearly perturbing the redox couple, the Fe^{+2}/Fe^{+3} in the molecular monolayer) and doing this in different frequencies in a single probe format as indicated in Figure 2*b*.

Therefore, the duration of the redox process, characterized by perturbing the electrons from metallic probe to redox states in the molecule, can be treated equivalently as the duration of a collision in the collision theory (the mathematical treatment is equivalent since the collision theory is the precursor theory for the collision frequency in the chemical reactions). This approach was first introduced by Markus Buttiker to describe the concept of mesoscopic capacitance.[6] In following the seminal works of Eisenbud[15], Wigner[16] and Smith[17] it has been noted that in collision theory the lifetime matrix ($\boldsymbol{Q}$) is directly correlated with scattering matrix ($\boldsymbol{S}$). The problem is treated quantum mechanically, using steady-state wave functions; the average time of residence in a region is the integrated density divided by the total flux in (or out), and the lifetime is defined as the difference between these residence times with and without interaction and the transformation properties require construction of the lifetime matrix given by

$$\boldsymbol{Q} = -i\hbar\boldsymbol{S}^{\dagger}\frac{d\boldsymbol{S}}{d\bar{\mu}} \tag{SI. 8}$$

In general, when a multiple solution of the Schrödinger equation exists for the energy variation associated with a single electron transfer/transport, $\bar{\mu} = -eV$, transformation requirements lead to the matrix $\boldsymbol{Q}$, where the diagonal elements, Q_{ii}, are the lifetimes associated with the particular solution of the Schrödinger equation ψ_i defined by an incoming wave in the ith channel. Apparently different is the consideration by Eisenbud[15] and Wigner[16] of a wave-packet analysis. In the case of scattering, which can be described by a simple phase-shift, ϕ, they show that a suitable definition of a delay-time (in our particular case the delay of the current response when linearly perturbing an stationary potential state of the molecular film) involves the energy derivative of the phase-shift as

$$\Delta t = -i\hbar\boldsymbol{s}^{\dagger}\frac{d\boldsymbol{s}}{d\bar{\mu}} \tag{SI. 9}$$

which was proved by Smith to be exactly the same as the lifetime Q_{ii}. This proof provides the clue to a general relationship between the scattering matrix $\boldsymbol{S}$ and the lifetime matrix $\boldsymbol{Q}$. If $\boldsymbol{S}$ is written as $\boldsymbol{s} = e^{i\phi}$ then

$$\boldsymbol{Q} = -i\hbar\boldsymbol{s}^{\dagger}\frac{d\boldsymbol{s}}{d\bar{\mu}} = \hbar\frac{d\phi}{d\bar{\mu}} \tag{SI. 10}$$

This is a generalized expression and $\boldsymbol{S}$ is the scattering matrix for elastic or inelastic collision and $\boldsymbol{Q}$ is the lifetime matrix no matter the process is elastic or inelastic and was proved by Smith[17] in the space (in the one-dimension case). The case we are working on is exactly the one-dimension accordingly as presented in Figure 2b and as shown in the main text, in the context of molecular electrochemistry, we have considered that this situations is governed by an RC circuit where $\tau = R_q C_r$ and then

$$\tau = R_q C_r = \hbar\frac{d\phi}{d\bar{\mu}} \tag{SI. 11}$$

where R_q is the relaxation resistance as discussed and introduced in the main text and C_r is the redox capacitance as discussed in previous works.[1-4]

SI. 2.3. Electron Density and Capacitance

Very recently we have shown the relationship between capacitance spectroscopy (CS) and density function theory (DFT).[5] In DFT, the fundamental variable associated with all observables is, of course, the electron density, i.e. $\rho = \sum_i {\psi_i}^2$, where ψ_i can be expressed as a linear combination of Kohn-Sham orbitals. Therefore, in considering the mathematical methodology associated with Kohn-Sham formulation of finite-temperature density function theory,[18] the energy defining equations arise as[19]

$$H_{KS}\psi_i(\vec{r}) = \varepsilon_i\psi_i(\vec{r}) \qquad \text{(SI. 12)}$$

where H_{KS} is the Kohn-Sham Hamiltonian, $\psi_i(\vec{r})$ express the linear combination of Kohn-Sham orbitals and ε_i is the eigenvalue of that state. Eqn. (SI. 12) is related to the electron density by[19]

$$\rho(\vec{r}) = \sum_i |\psi_i(\vec{r})|^2 f \qquad \text{(SI. 13)}$$

where f is the Fermi-Dirac function as defined previously in section 1.2. In this context, the electron density (which for a molecular redox film is equivalent to the number of redox states per volume), $\rho = N_r/\Omega$ (where Ω is the volume), in the molecular film, can be calculated from the integral of DOS function $[f g_r(\bar{\mu}) = f C_r(\bar{\mu})/e^2]$ in the molecular film [noting that C_r in the context of Eqn. (SI. 13) is given by unit of the volume of the molecular film] as

$$\rho = \int_{-\infty}^{\infty} \frac{g_r(\bar{\mu})}{1+\exp[(\bar{\mu}-E_r)/k_B T]} d\bar{\mu} = \int_{-\infty}^{\infty} f \frac{C_r(\bar{\mu})}{e^2} d\bar{\mu} \qquad \text{(SI. 14)}$$

i.e., effectively by considering the shape of redox capacitance per unit of volume of the molecular film, C_r. This electrochemical/redox capacitance is a function of $\bar{\mu}$, i.e. the electrochemical of electrons in the metallic states. Note also that $\bar{\mu}$ is related to a change in electrochemical potential (dV) of the electrode by $d\bar{\mu} = -edV$ as mentioned in section 1.2.

Remembering that $g_r(-eV) = -dN_r/edV$ (see section 1.2). The corresponding version of Eqn. (S1. 14) to calculate the number of electrochemistry states, N_r, directly from electrochemical capacitance as a function of potential at finite temperatures is

$$N_r = \int_{-\infty}^{\infty} \frac{e g_r(eV)}{1+\exp[(eV-E_r)/k_B T]} dV = \int_{-\infty}^{\infty} f[C_r(V)/e] dV \qquad \text{(SI. 15)}$$

Therefore, practically N_r is a quantity easily obtained by the integral of redox capacitance as a function of electrochemical potential of the electrode as indicated in Figure 4*a* of the main text. The obtained pattern is a Gaussian shape indicated as discussed in previous works.[1-5] Note that in the zero temperature limit $C_r(\bar{\mu}) = e^2 g_r(\bar{\mu})$ so that $N_r = \int C_r(\bar{\mu}) d\bar{\mu} = \int e^2 g_r(\bar{\mu}) d\bar{\mu}$ as indicated in the main text.

SI. 2.4. Electrochemical States (or Density) and the Quantum Transport

In considering the redox states coupled to metallic single probe are continuum it can be observed that the redox charge is coupled to the redox capacitance by

$$dq_r = C_r dV \quad \text{(SI. 16)}$$

where q_r is the charge associated with the redox process (redox reaction) only. In considering infinitesimal variation in time (at steady-state condition) of Eqn. (SI. 16) we obtain $dq_r/dt = C_r dV/dt$ which is equivalent to $di_r/dt = C_r dV/dt$, where i_r is the redox current (faradaic only[2,5,20]). In adopting $d\bar{\mu} = -edV$ and with some rearrangement Eqn. (SI. 16) turns into

$$ei_r = -C_r \frac{d\bar{\mu}}{dt} \quad \text{(SI. 17)}$$

If we note now that for a given charge transfer event there is some quantum uncertain ruled by uncertainty principle[21] $dEdt = h/2$, where $dE = -ed\mathrm{V}$ then $1/dt = -(2ed\mathrm{V})/h$ and substituting in Eqn. (SI. 17) and rearranging we obtain

$$i_r = \frac{2C_r}{h} d\mathrm{V} d\bar{\mu} \quad \text{(SI. 18)}$$

Now if we differentiate both side of Eqn. (SI. 18) as a function of potential we have the (redox) conductance

$$dG(\bar{\mu}) = \frac{di_r}{dV} = \frac{2C_r}{h} d\bar{\mu} \quad \text{(SI. 19)}$$

Since $C_r(\bar{\mu}) = e^2 dN_r/d\bar{\mu}$ by definition[1,2,5] and substituting in Eqn. (SI. 19) we obtain

$$dG(\bar{\mu}) = \frac{di_r}{dV} = \frac{2e^2}{h} dN_r = G_0 dN_r \quad \text{(SI. 20)}$$

Finally, by integrating Eqn. (SI. 20) we have the total quantum of conductance (considering the zero temperature limit) written as a function of the redox density of states

$$G = G_0 \int C_r(\bar{\mu}) d\bar{\mu} = G_0 N_r \quad \text{(SI. 21)}$$

And by comparison with Eqn. (2) and Eqn. (4) it can be noted that in an ensemble of continuum quantum channels $N_r = \int C_r d\bar{\mu}$ the conductance is proportional to the density of quantum/redox channels. Precisely, for a continuum redox density of states (as is the case of the molecular junctions studied in this work[5]), this is equivalent to assume that each redox state is associated with a quantum conducting channel (bridging the redox molecular states and those of the metallic probe).

In summary, the redox/electron density obtained in the electrochemistry of molecular (single probe) junctions is equivalent to the conductance through by considering the charge transfer rate involved and connecting the metallic states to those of redox states.

References

1 Bueno, P. R., Mizzon, G. & Davis, J. J. Capacitance Spectroscopy: A Versatile Approach To Resolving the Redox Density of States and Kinetics in Redox-Active Self-Assembled Monolayers. *Journal of Physical Chemistry B* **116**, 8822-8829, doi:10.1021/jp303700f (2012).

2 Santos, A., Carvalho, F. C., Roque-Barreira, M.-C. & Bueno, P. R. Impedance-derived electrochemical capacitance spectroscopy for the evaluation of lectin-glycoprotein binding affinity. *Biosensors & Bioelectronics* **62**, 102-105, doi:10.1016/j.bios.2014.06.034 (2014).

3 Bueno, P. R. & Davis, J. J. Elucidating redox level dispersion and local dielectric effects within electroactive molecular films. *Anal. Chem.* **86**, 1977-2004, doi:10.1021/ac4031708 (2014).

4 Bueno, P. R., Fabregat-Santiago, F. & Davis, J. J. Elucidating Capacitance and Resistance Terms in Confined Electroactive Molecular Layers. *Analytical Chemistry* **85**, 411-417, doi:10.1021/ac303018d (2013).

5 Bueno, P. R., Gustavo, T. F. & Davis, J. J. Capacitance Spectroscopy and Density Functional Theory. *Physical Chemistry Chemical Physics*, doi:10.1039/c4cp06015f (2015).

6 Büttiker, M., Thomas, H. & Prêtre, A. Mesoscopic capacitors. *Phys. Lett. A* **180**, 364-369 (1993).

7 Luryi, S. Quantum capacitance devices. *Applied Physics Letters* **52**, 501 (1988).

8 Fernandes, F. C. B., Goes, M. S., Davis, J. J. & Bueno, P. R. Label free redox capacitive biosensing. *Biosensors & Bioelectronics* **50**, 437-440, doi:10.1016/j.bios.2013.06.043 (2013).

9 Fernandes, F. C. B., Santos, A., Martins, D. C., Goes, M. S. & Bueno, P. R. Comparing label free electrochemical impedimetric and capacitive biosensing architectures. *Biosensors & Bioelectronics* **57**, 96-102, doi:10.1016/j.bios.2014.01.044 (2014).

10 Lehr, J., Hobhouse, G. C., Fernandes, F. C. B., Bueno, P. R. & Davis, J. J. Label-free Capacitive Diagnostics: Exploiting Local Redox Probe State Occupancy (vol 86, pg 2559, 2014). *Analytical Chemistry* **86**, 3682-3682, doi:10.1021/ac500788e (2014).

11 Heikkilä, T. T. *The Physics of Nanoelectronics*. (Oxford University Press, 2013).

12 Landauer, R. Electrical resistance of disordered one-dimentional lattices. *Philosophical Magazine* **21**, 863 (1970).

13 Buttiker, M., Imry, Y., Landauer, R. & Pinhas, S. Generalized many channel conductance formula with application to small rings. *Physical Review B* **31**, 6207 (1985).

14 Landauer, R. Spatial Variation of Currents and Fields Due to Localized Scatterers in Metallic Conduction. *IBM Journal of Research and Development* **1**, 223-231 (1957).

15 Eisenbud, L. PhD thesis, Princeton, (1948).

16 Wigner, E. P. Lower limit for the energy derivative of the scattering phase shift. *Physical Review* **98**, 145-147 (1955).

17 Smith, F. T. Lifetime matrix in collision theory. *Physical Review* **118**, 349-356, doi:10.1103/PhysRev.118.349 (1960).

18 Car, R. & Parrinello, M. Unified approach for molecular-dynamics and density-functional theory. *Physical Review Letters* **55**, 2471-2474, doi:10.1103/PhysRevLett.55.2471 (1985).

19 Parr, R. G. & Weitao, Y. *Density-Functional Theory of Atoms and Molecules*. (Oxford Science Publication, 1994).

20 Goes, M. S., Rahman, H., Ryall, J., Davis, J. J. & Bueno, P. R. A Dielectric Model of Self-Assembled Monolayer Interfaces by Capacitive Spectroscopy. *Langmuir* **28**, 9689-9699, doi:10.1021/la301281y (2012).

21 Landau, L. D. & Lifshitz, E. M. *Quantum mechanics: non-relativistic theory*. (Pergamon Press, 1977).
